# Image-guided study of inter-fraction and intra-fraction set-up variability and margins in reverse semi-decubitus breast radiotherapy

**DOI:** 10.1186/s13014-018-1200-1

**Published:** 2018-12-27

**Authors:** Jie Lee, Shih-Hua Liu, Jhen-Bin Lin, Meng-Hao Wu, Chieh-Ju Wu, Hung-Chi Tai, Shih-Ming Hsu, Yin-Ju Chen, Jo-Chiao Tai, Yu-Jen Chen

**Affiliations:** 1 Department of Radiation Oncology, MacKay Memorial Hospital, 92, Section 2, Chung-Shan North Road, Taipei, 104 Taiwan; 2 Department of Medicine, MacKay Medical College, New Taipei City, Taiwan; 3 Department of Biomedical Imaging and Radiological Sciences, National Yang-Ming University, No. 155, Sec. 2, Li-Nong St., Beitou District, Taipei, 112 Taiwan, Republic of China; 4 Department of Radiation Oncology, Changhua Christian Hospital, Changhua, Taiwan

**Keywords:** Reverse semi-decubitus technique, Inter-fraction reproducibility, Intra-fraction motion, Breast radiotherapy

## Abstract

**Background:**

This study aimed to evaluate the inter-fraction set-up error and intra-fraction motion during reverse semi-decubitus (RSD) breast radiotherapy, and to determine a planning target volume (PTV) margin.

**Material and methods:**

Pre- and post-treatment cone-beam computed tomography (CBCT) scans were prospectively acquired at fractions 1, 4, 7, 8, 11, and 14 for 30 patients who underwent RSD breast radiotherapy. Online correction for initial set-up error greater than 5 mm or 2° was performed and post-correction CBCT was acquired. An off-line analysis was performed to quantify initial and residual inter-fraction set-up errors and intra-fraction motion in three-dimensions. Patient inter-fraction errors were analysed for time trends during the course of radiotherapy. PTV margins were calculated from the systematic and random errors.

**Results:**

The initial inter-fraction population systematic errors were 1.8–3.3 mm (translation) and 0.5° (rotation); random errors were 1.8–2.1 mm (translation) and 0.3–0.5° (rotation). After online correction, the residual inter-fraction population systematic errors were 1.2–1.8 mm (translation) and 0.3–0.4° (rotation); random errors were 1.4–1.6 mm (translation) and 0.3–0.4° (rotation). Intra-fraction population systematic and random errors were ≤ 1.3 mm (translation) and ≤ 0.2° (rotation). The magnitude of inter-fraction set-up errors in the anterior-posterior direction, roll, and yaw were significantly correlated with higher body weight and body mass index (BMI). The inter-fraction set-up error did not change significantly as a function of time during the course of radiotherapy. The magnitude of intra-fraction motion was not correlated with patient characteristics and treatment time. The total PTV margins accounting for pre-correction and intra-fraction errors were 6.5–10.2 mm; those accounting for post-correction and intra-fraction errors were 4.7–6.3 mm.

**Conclusions:**

CBCT is an effective modality to evaluate and improve the inter-fraction set-up reproducibility in RSD breast radiotherapy, particularly for patients with higher BMI. Intra-fraction motion was minimal during RSD breast radiotherapy.

## Introduction

Several studies have shown an increase in the rate of ischaemic heart disease after adjuvant radiotherapy for left-sided breast cancer [1–4]. The occurrences of acute coronary events exhibit dose-effect relationships [3–5]. To improve the therapeutic ratio of breast radiotherapy, efforts have been made to develop simulation and treatment techniques in order to reduce the volume of cardiac irradiation.

Deep inspiration breath-hold (DIBH) and prone are widely used to improve cardiac dosimetry [6–15]. However, certain factors may preclude their use, such as intolerance of the technique by the patient or requirement for specialized equipment. The reasons why patients may not be able to tolerate breath-hold include medical co-morbidity, anxiety, inability to tolerate specialized equipment, or language barriers. The benefits of the prone technique in cardiac sparing might be limited to large breasted women, and may in fact be detrimental in women with small breasts [15–18]. A novel free-breathing technique for left breast irradiation in the reverse semi-decubitus (RSD) position could benefit patients unable to tolerate breath-hold, by reducing the cardiac dose [19, 20]. The RSD simulation was performed by rotating the patient into a semi-lateral decubitus position, with the right side towards the treatment couch and the left side elevated. To ensure that radiotherapy is given in a safe and consistent manner, the reproducibility of the RSD, particularly that of rotation, is of great importance. However, the translational and rotational inter-fraction set-up error and intra-fraction motion are unknown. Cone-beam computed tomography (CBCT) can help reduce the setup error and random deviation, by quantifying the three-dimensional translational and rotational errors [21, 22]. This study aimed to evaluate the translational and rotational inter-fraction set-up error and intra-fraction motion using CBCT and calculate an appropriate clinical target volume (CTV) to planning target volume (PTV) margin for RSD breast radiotherapy.

## Materials and methods

### Patients

This study was approved by the Institutional Review Board and all procedures were performed according to the Declaration of Helsinki. Thirty patients with early-stage left-sided breast cancer were enrolled at our institutions. All patients had undergone breast-conserving surgery, followed by left breast radiotherapy, between September 2017 and January 2018.

### Patient positioning and image acquisition

**Fig. 1:**
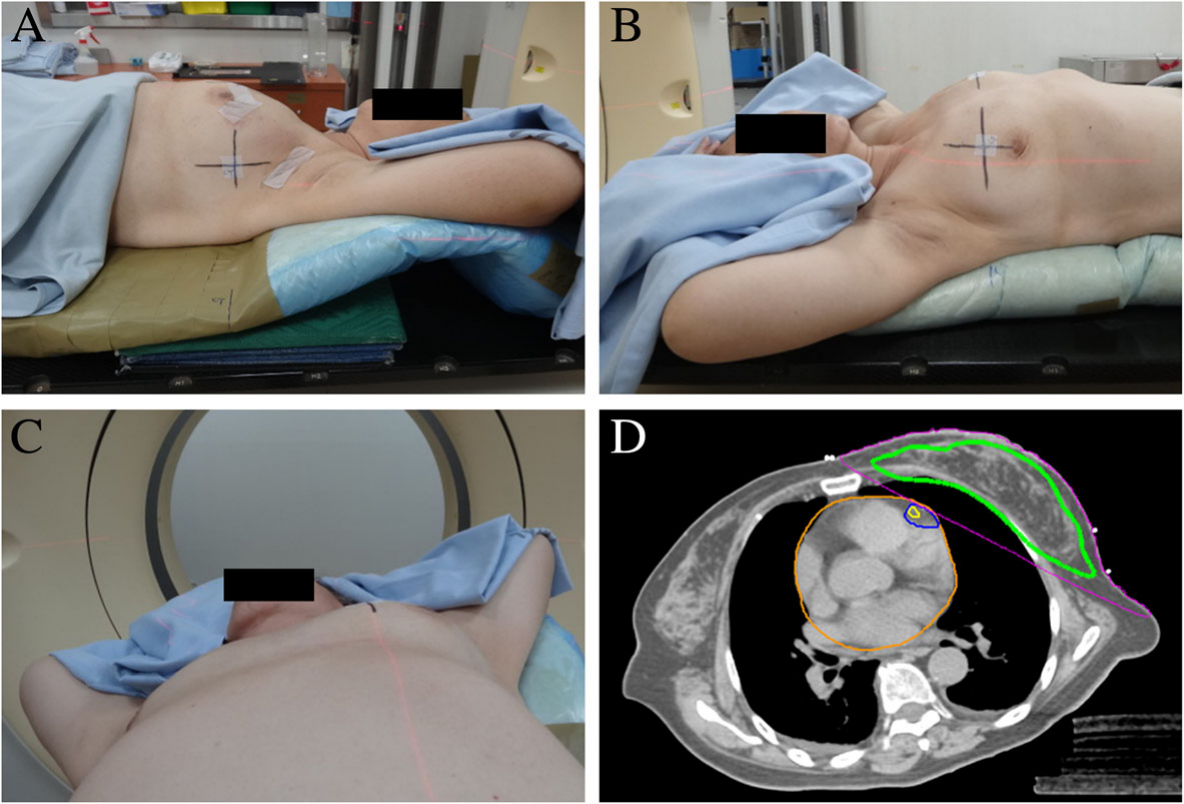
This patient was simulated in the reverse semi-decubitus position (A-C). The patient was rotated to the right side, and the arms were abducted by 70–110 degrees. The upper torso was elevated slightly on a customized breast-board, and the patient was immobilized by using an Alpha Cradle. The axial RSD CT planning images for this patient, using tangent fields, are shown in (D). Displayed isodoses are 4000 cGy (green) and 2000 cGy (purple). The left anterior descending coronary artery (LAD), LAD region, and heart are outlined in yellow, blue, and orange, respectively

All patients undergoing breast radiotherapy were scanned in the RSD position on the custom-made Alpha Cradle (Fig. 1). RSD simulation was performed by rotating the patient into a semi-lateral decubitus position, with the right side towards the treatment couch and the left side elevated [19, 20]. The patient’s arms were abducted above the head and immobilized using an Alpha Cradle. Markers were placed ipsilaterally, 2 cm lateral to all palpable breast tissue along the midaxillary line and midsternal line. Surgical scars and all visible breast tissue were circled with wires. Patients were scanned using the Philips Brilliance CT Big Bore scanner (Philips Healthcare, Amsterdam, Netherlands). The CT images were acquired from the C6 vertebral body to the diaphragm at 3-mm slice intervals. The simulation and treatment were performed while free-breathing.

### Treatment

Figure 1d shows the axial RSD CT planning images. The CTV encompassed the breast tissue visualized on CT. The CTV was limited by the pectoral fascia. The PTV was generated by adding a 7-mm margin around the CTV, except at the skin surface. Mammary chain or supraclavicular fossa irradiation was not performed. The organs at risk included the heart, left anterior descending coronary artery (LAD), and LAD region, which were delineated based on the previously published cardiac atlas [23–25]. All plans were implemented using a three-dimensional treatment planning system (Pinnacle, version 9.8; Phillips Medical Systems, Fitchburg, WI, USA). Patients were treated using 6–10 MV photons with 40 Gy in 15 fractions over 3 weeks. The tangential wedge-based field plans were applied, and ≥ 90% of the whole breast CTV was encompassed by the 95% isodose.

### CBCT analysis

The RSD position was reproduced during treatment by aligning skin tattoos to lasers, with photographs used for assistance where necessary. The CBCT images of the chest were acquired before (pre-treatment CBCT) and after (post-treatment CBCT) treatment at fractions 1, 4, 7, 8, 11, and 14, by using the Elekta Versa-HD X-ray Volume Imaging system (Elekta, Crawley, UK). The pre-treatment CBCT data were registered online to the treatment planning CT. If there was rotational error greater than 2°, the patient was manually repositioned and another CBCT scan was acquired. Translational displacements of greater than 5 mm were corrected online by using automated treatment table movement; then, a second CBCT was performed to confirm the accuracy of correction.

All CBCT data were analysed offline. Automated rigid registration was implemented by using the Pinnacle, with priority given to accurate registration of the sternum and ribs on the left side to achieve a position that would provide the most accurate bony reproducibility. The displacement of the isocentre was calculated in the three axes (RL: right–left; AP: anterior–posterior; and SI: superior–inferior). The + and – signs indicate right/anterior/superior and left/posterior/inferior directions, respectively. The nomenclature used in this study was as follows: roll is a rotation about the SI axis, yaw is a rotation about the AP axis, and pitch is a rotation about the RL axis. The pre-correction CBCT scan acquired after the in-room setup was used to calculate the initial inter-fraction error. The post-correction CBCT scan acquired after any corrections for translational or rotational set-up error, or the pre-correction CBCT scan for fractions where the initial setup was within ±5 mm set-up error, was used to calculate the residual inter-fraction error. The difference between the pre-correction and post-treatment CBCT was used to calculate intra-fraction motion for patients with initial set-up without correction; the post-correction and post-treatment CBCT was used for intra-fraction motion analysis for patients with correction for translational or rotational set-up error, as previously mentioned. The data are presented as the mean ± standard deviation (SD).

### Predictive factors for the inter-fraction set-up error and intra-fraction motion

Patient characteristics, such as age, body weight, and body mass index (BMI) were recorded to examine predictive factors for inter-fraction set-up error and intra-fraction motion. Correlations between set-up errors and patient body characteristics were analysed by using Pearson’s product-moment correlation coefficient (R), and the t-test was used to test the significance of the correlation. The treatment time was defined as the time interval between pre-correction and post-treatment CBCT acquisition time for patients with initial set-up without correction; the time interval between post-correction and post-treatment CBCT acquisition time was used for patients having correction for translational or rotational set-up error. The correlation between treatment time and intra-fraction motion was also analysed. One-way repeated measures analysis of variance (ANOVA) was used to analyse the time dependency of inter-fraction set-up errors. The significance level was set to < 0.05. The patients were divided into two groups on the basis of the median values of factors with significance, and the Student’s t-test was used to analyse differences between the groups.

### Calculation of PTV margin

Mean displacements and SD were calculated in three-dimensions for each patient. Population systematic errors (Σ) were calculated from the SD of all mean displacements, and population random errors (σ) from the root mean square of all SD values [26]. The CTV–PTV margins were calculated based on the van Herk formula: 2.5Σ + 0.7σ [27]. The overall Σ and σ were defined as the square root of the quadratic sum of the inter-fraction and intra-fraction Σ and σ, respectively [26].

## Results

The median (range) age, body weight, and BMI were 52 (38–75) years, 63.0 (47.0–88.2) kg, and 25.9 (18.1–33.6) kg/m^2^, respectively. A total of 435 CBCT images were acquired from 30 left breast cancer patients, including 180 pre-correction images, 75 post-correction images, and 180 post-treatment images. Among all patients, the median number of fractions with set-up errors requiring correction and re-imaging was two (range, 0–5). When dividing patients into two groups on the basis of the median value of BMI, the median numbers of fractions with set-up errors requiring correction and re-imaging were zero (range, 0–3) and four (range, 1–5) for patients with ≤25.9 kg/m^2^ and > 25.9 kg/m^2^, respectively (*p* < 0.001).

### Inter-fraction set-up error

**Fig. 2:**
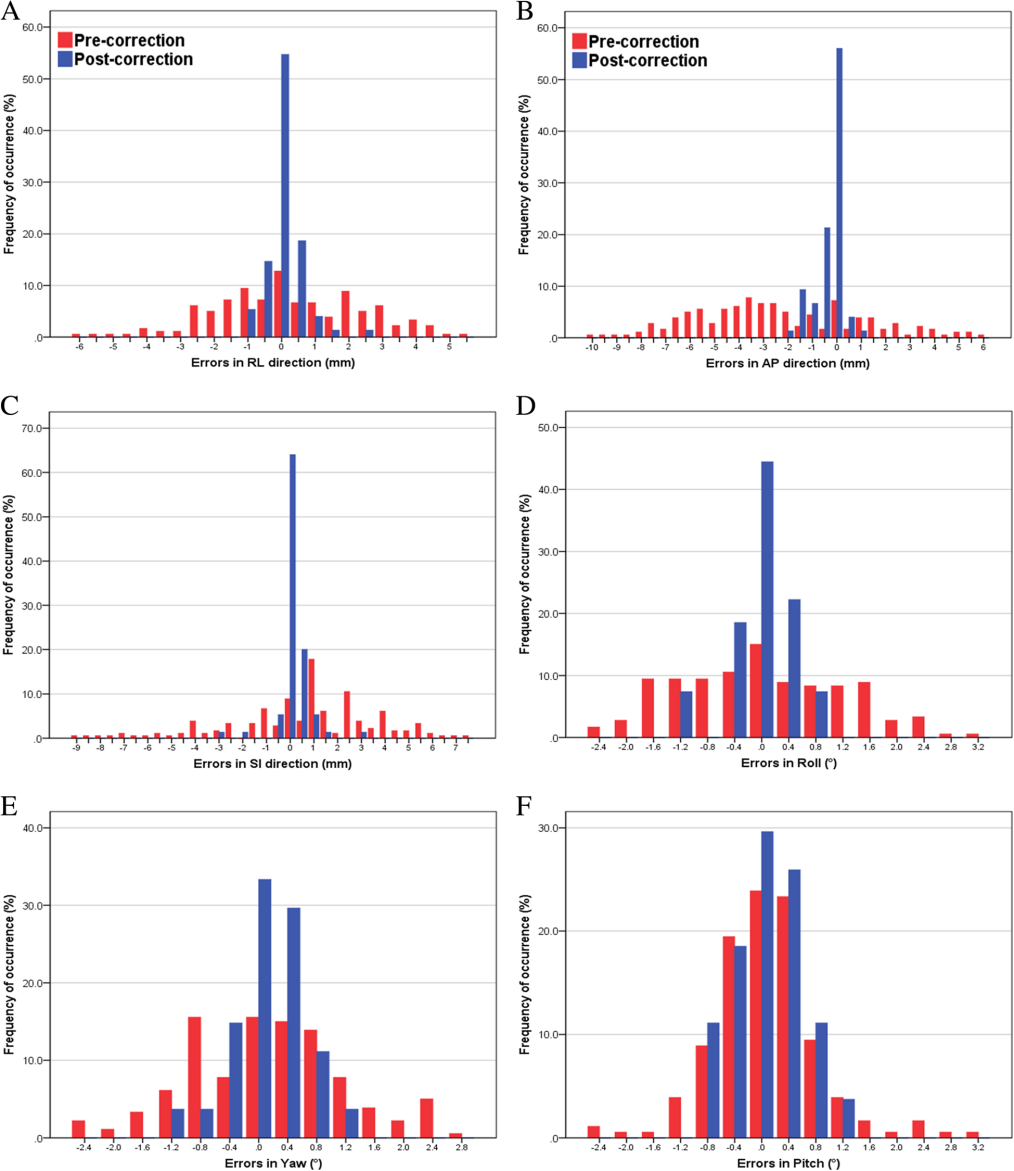
Distribution of differences between pre- or post-correction cone-beam computed tomography (CT) scans and planning CT scans. Translational set-up errors are shown in (**a**), (**b**), and (**c**) for the right–left, anterior–posterior, and superior–inferior directions, respectively. Rotational set-up errors are shown in (**d**), (**e**), and (**f**) for the roll, yaw, and pitch, respectively. RL, right–left; AP, anterior–posterior; and SI, superior–inferior

The distributions of inter-fraction setup errors in each of the three orthogonal directions were calculated by using the 180 pre-correction images and 75 post-correction images (Fig. 2). For initial inter-fraction shifts, the numbers of fractions exceeding ±5 mm in the RL, AP, and SI directions were 5 (2.7%), 51 (28.3%) and 25 (13.9%), respectively; 16 (8.9%), 14 (7.8%) and 8 (4.4%) fractions exceeded 2° for roll, yaw, and pitch, respectively. In all directions, the distributions obtained from the post-correction scans were narrower than those obtained from the pre-treatment scans, all lying within the 5 mm and 2° tolerance level. Table 1 displays the population mean, systematic, and random inter-fraction translational and rotational errors. The residual inter-fraction Σ and σ values were smaller than the initial inter-fraction Σ and σ values.

**Table 1:** Population mean (M), systematic (Σ), and random (σ) translational and rotational errors in 3-dimensions in reverse semi-decubitus positions

		RL (mm)	AP (mm)	SI (mm)	Roll (°)	Yaw (°)	Pitch (°)
Initial inter-fraction error	M	0.2	−2.5	0.7	1.1	1.0	0.7
	Σ	1.8	3.3	2.7	0.5	0.5	0.5
	σ	1.8	1.8	2.1	0.5	0.4	0.3
Residual inter-fraction error	M	0.2	−0.9	0.4	1.0	0.9	0.6
	Σ	1.2	1.8	1.4	0.4	0.4	0.3
	σ	1.4	1.5	1.6	0.4	0.4	0.3
Intra-fraction motion	M	−0.1	−0.4	0.2	0.2	0.2	0.2
	Σ	0.9	1.0	1.0	0.1	0.1	0.1
	σ	1.0	1.3	1.2	0.2	0.1	0.1

*RL* right-left direction, *AP* anterior–posterior direction, *SI* superior-inferior direction

**Fig. 3:**
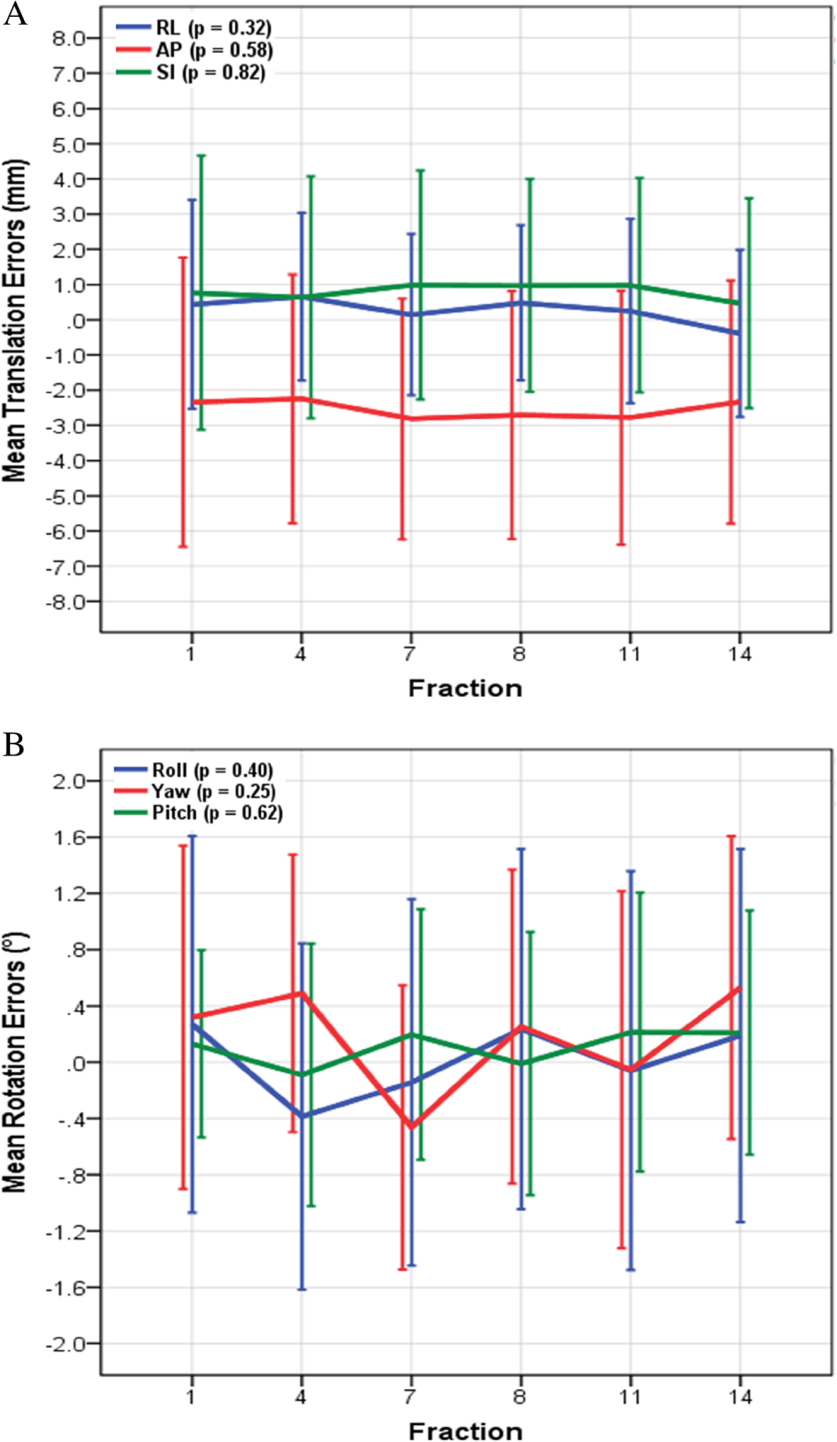
Inter-fraction translational (**a**) and rotational (**b**) set-up errors were plotted as a function of time, in fractions. Shown in the legend are the *p* values from repeated measures ANOVA. Error bars indicate 1 standard deviation

The magnitude of the inter-fraction set-up errors in the AP direction, roll, and yaw were correlated with high body weight and BMI (Table 2). The inter-fraction translational and rotational set-up errors did not change significantly as a function of time (Fig. 3). When dividing the patients into two groups based on the median BMI, the mean set-up errors were − 1.1 ± 3.0 and − 4.0 ± 2.9 mm in the AP direction (*p* = 0.01), 0.7° ± 0.3° and 1.5° ± 0.5° in the roll (*p* < 0.001), and 0.8° ± 0.4° and 1.1° ± 0.6° in the yaw (*p* = 0.08), for patients with BMI ≤ 25.9 kg/m^2^ and > 25.9 kg/m^2^, respectively. The inter-fraction set-up errors were − 1.6 ± 2.6 and − 3.4 ± 3.7 mm in the AP direction (*p* = 0.14), 0.9° ± 0.5° and 1.4° ± 0.5° in the roll (*p* = 0.004), and 0.8° ± 0.4° and 1.2° ± 0.6° in the yaw (*p* = 0.06), for patients with weight ≤ 63 kg and > 63 kg, respectively.

**Table 2:** Correlation coefficients between patient characteristics and translational and rotational set-up errors

Characteristics	Median (range)	Inter-fraction^a^						Intra-fraction					
		RL	AP	SI	Roll	Yaw	Pitch	RL	AP	SI	Roll	Yaw	Pitch
Age	52 (38–75)	0.17	0.05	0.09	0.28	0.02	0.05	0.29	0.07	0.01	0.22	0.07	0.13
Body weight (kg)	63.0 (47.0–88.2)	0.33	0.54†	0.13	0.54†	0.50†	0.30	0.02	0.33	0.30	0.24	0.21	0.22
BMI (kg/m^2^)	25.9 (18.1–33.6)	0.24	0.55†	0.12	0.68†	0.53†	0.33	0.03	0.23	0.32	0.28	0.24	0.13
Treatment time (min)	4.1 (3.0–5.6)	–	–	–	–	–	–	0.08	0.32	0.10	0.14	0.03	0.14

*RL* right-left direction, *AP* anterior–posterior direction, *SI* superior-inferior direction, *BMI* body mass index

†Correlation was significant at *p* < 0.05

^a^The pre-correction cone-beam computerized tomography images were used for these analyses

### Intra-fraction motion

**Fig. 4:**
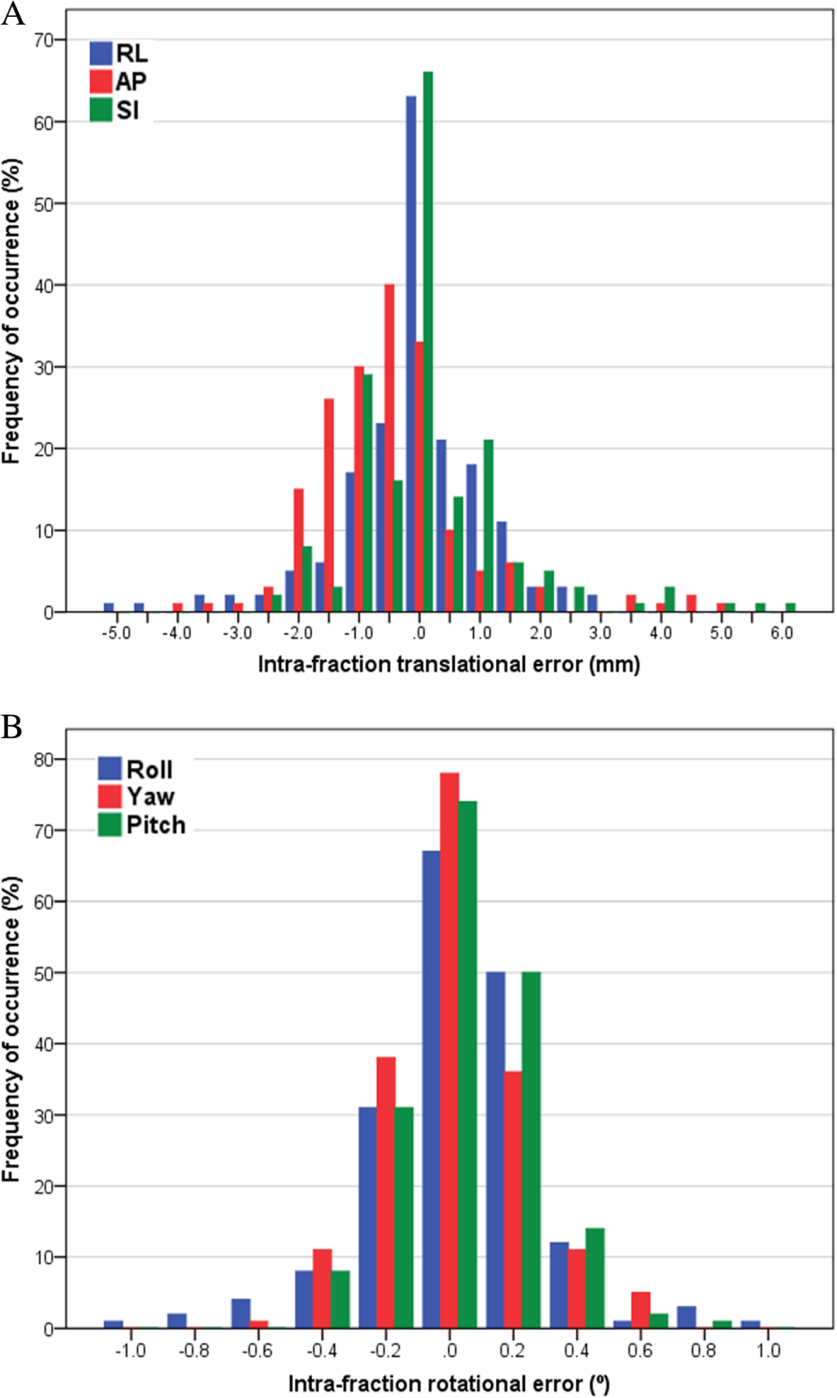
Histogram distribution of intra-fraction translational (**a**) and rotational (**b**) motion

The mean time interval of intra-fraction motion assessment was 4.1 ± 0.8 min (range, 3.0–5.6 min). Figure 4 shows a histogram distribution of intra-fraction motion. The numbers of fractions exceeding ±5 mm in the RL, AP, and SI directions were 1 (0.6%), 1 (0.6%) and 3 (1.7%), respectively. The intra-fraction rotational errors were all within 1°. Table 1 displays the population mean systematic and random intra-fraction translational and rotational errors. The magnitude of intra-fraction translational and rotational motion was not correlated with age, body weight, BMI, or treatment time (Table 2).

### PTV margins for reverse semi-decubitus breast radiotherapy

For all patients, a comparison of the PTV margins obtained from pre-correction, post-correction, and post-treatment CBCT images showed that PTV margins can be reduced with the online correction (Table 3). The total margins accounting for initial inter-fraction set-up error and intra-fraction motion were 6.5, 10.2, and 8.9 mm in the RL, AP, and SI directions, respectively; in contrast, the corresponding required target margins were only 4.7, 6.3, and 5.4 mm in each of the three directions, if the residual and intra-fraction errors were considered after online correction.

**Table 3:** Calculated CTV–PTV margins in 3-dimensions

Margin	RL (mm)	AP (mm)	SI (mm)
Initial inter-fraction			
Overall	5.8	9.4	8.3
BMI > 25.9 kg/m^2^	5.9	9.8	8.3
BMI ≤25.9 kg/m^2^	5.4	7.4	8.2
Residual inter-fraction			
Overall	4.0	5.5	4.7
BMI > 25.9 kg/m^2^	3.1	4.4	3.7
BMI ≤25.9 kg/m^2^	4.8	6.4	5.6
Intra-fraction			
Overall	2.9	3.3	3.4
BMI > 25.9 kg/m^2^	2.9	3.3	3.1
BMI ≤25.9 kg/m^2^	3.0	3.2	3.7
Total without CBCT correction			
Overall	6.5	10.2	8.9
BMI > 25.9 kg/m^2^	6.6	10.3	8.8
BMI ≤25.9 kg/m^2^	6.3	8.3	9.1
Total with CBCT correction			
Overall	4.7	6.3	5.4
BMI > 25.9 kg/m^2^	4.3	5.4	4.7
BMI ≤25.9 kg/m^2^	5.6	7.2	6.8

*CTV* clinical target volume, *PTV* planning target volume, *RL* right-left direction, *AP* anterior–posterior direction, *SI* superior-inferior direction, *BMI* body mass index, *CBCT* cone-beam computerized tomography

For patients with higher BMI, the calculated PTV margins for initial inter-fraction set-up error and intra-fraction motion were larger than those for patients with lower BMI (Table 3). With online correction, the PTV margins could be reduced both for patients with BMI ≤ 25.9 kg/m^2^ and > 25.9 kg/m^2^.

## Discussion

This is the first study to analyse inter-fraction set-up error and intra-fraction motion by CBCT scans for RSD breast radiotherapy. The CBCT allows a reduction in inter-fraction set-up errors, particularly for heavier patients. The magnitude of inter-fraction set-up errors in the AP direction, roll, and yaw were significantly correlated with higher body weight and BMI. Intra-fraction motion was small, suggesting that patients were able to perform a stable RSD within the treatment fraction.

In the present study, the RSD position was reproduced by aligning skin tattoos to lasers; the set-up variability was evaluated by analysing CBCT results. Pre-treatment CBCT could effectively detect the translational and rotational set-up errors that required adjustment and improved the accuracy of RSD breast radiotherapy, particularly for patients with higher BMI. By using image-guided set-up, the inter-fraction systemic errors were reduced from 1.8–3.3 mm to 1.2–1.8 mm. Most of the patients had inter-fraction set-up error toward the posterior direction (Fig. 2b); the magnitude of set-up errors in the AP direction was significantly correlated with high weight and BMI. The inter-fraction rotational errors in the roll and yaw were also significantly larger for heavier patients. These findings might be attributed to differences in the magnitude of compression of the subcutaneous back fat in each fraction [28], although set-up included matching to skin tattoos. In addition, the inter-fraction set-up error did not change significantly as a function of time in this study. Image-guided set-up would be helpful to ensure favourable reproducibility in RSD breast radiotherapy.

Intra-fraction motions were evaluated by comparing pre-treatment and post-treatment CBCT without considering respiratory-induced motion in this study. This implied that only intra-fraction baseline drift, which may depend on the deformation or movement of fat or muscle relaxation during treatment, could be evaluated. We found that intra-fraction baseline drift, in terms of translation and rotation, was small during RSD breast radiotherapy (Table 1); this suggested that patients could maintain the RSD position with high stability during radiotherapy. The clinical and dosimetric impact of such minimal intra-fraction motion might be limited. In addition, baseline drift that occurs during prolonged treatment might induce notable uncertainties [29]. The patients in the present study received tangential radiotherapy with a mean treatment time of 4.1 ± 0.8 min; however, intra-fraction motion was not correlated with treatment time. The main intra-fraction baseline drifts were in the posterior direction; this finding was similar to the findings in two recent studies, where intra-fraction motion was evaluated in patients receiving supine breast radiotherapy [28, 29]. It might also be attributed to the compression of back subcutaneous fat or muscle relaxation during treatment [28]. However, the magnitude of intra-fraction translational and rotational motion was not correlated with body weight and BMI in this study.

The isotropic 7-mm CTV-PTV margin was used to treat patients in this study; a total of 19 out of 180 fractions (10.6%) showed an inter-fraction set-up error exceeding 7 mm. The frequencies of inter-fraction set-up errors exceeding 7 mm along the RL, AP, and SI axes were 1.1, 7.8, and 2.8%, respectively. Furthermore, most of the inter-fraction set-up errors exceeding 7 mm occurred in patients with a BMI > 25.9 kg/m^2^ (73.7%). An increase in PTV margins based on the van Herk formula using these data would ensure greater coverage of the target, but would also increase the radiation to nearby organs at risk. According to this study, the clinical benefit of increased PTV margins might be limited especially when tangential field radiotherapy was used. If the more conformal technique like intensity-modulated radiotherapy or volumetric arc therapy was used, substantial set-up errors would potentially have an impact on the total dose to the target and adjacent organs at risk, and these calculated margins might be used [30, 31]. Compared with the calculated margins for supine or prone position in the previous studies [10, 32], the calculated margins for RSD in this study were smaller. A possible explanation might be the differences in body composition between Western and Asian populations; the BMI of most Asian populations are lower than those of the North American population and possibly some European populations [33]. Online image guidance could help minimize the occurrence of substantial set-up error and potentially permit reduction in PTV margins [21]. The use of CBCT might be of greater benefit to patients with higher body weight or BMI. In the present study, the calculated PTV margins were reduced after image-guided set-up. However, we found that patients with BMI > 25.9 kg/m^2^ had greater reduction in PTV margins; this may have been related to the greater number of fractions requiring correction for set-up errors greater than 5 mm and small residual errors in these patients. In contrast, we found that most initial set-up errors were within the 5-mm tolerance setting in patients with BMI ≤ 25.9 kg/m^2^. Hence, the calculated PTV margin with CBCT correction was smaller for heavier patients in this study. In addition to CBCT, three-dimensional surface imaging or an optical tracking system might also be useful to compensate for the inter-fraction set-up errors and intra-fraction motion in breast radiotherapy, without the use of additional radiation [28, 29, 34]. However, the evaluation of set-up variability during RSD breast radiotherapy by three-dimensional surface imaging is beyond the scope of this study and must be addressed in future studies.

There are some limitations in this study. First, this study only evaluated six fractions among all 15 fractions of radiotherapy, rather than in the setting of daily CBCT. For the other nine fractions of radiotherapy, the RSD position was reproduced by aligning skin tattoos to lasers alone. Hence, substantial set-up errors might have occurred during these fractions, particularly for the heavier patients. A study with daily image-guided set-up would provide more comprehensive views of set-up errors and robust calculations of PTV margins in RSD breast radiotherapy. Second, this study only analysed patient set-up error; notably, changes in breast shape and reproducibility of the heart may play roles in RSD breast radiotherapy [31, 35]. Future studies evaluating the volumetric variation of the breast, as well as the reproducibility of the heart and LAD, are needed. Third, the respiratory-induced motion during RSD breast radiotherapy could not be evaluated in this study. Although previous studies have evaluated respiratory-induced motion in the supine or prone positions [10, 28], the optimal margin (including respiratory-induced motion in the RSD position) must be investigated in future studies. Despite these limitations, the present study is the first to evaluate inter-fraction set-up error and intra-fraction motion by using CBCT in RSD breast radiotherapy. RSD is an alternative technique for patients who are not able to tolerate breath-holding [19, 20]. At our institutions, RSD positioning is now one of the standard techniques because patients can receive breast radiotherapy with high comfort and compliance. Generally, the overall set-up and treatment time of RSD breast radiotherapy is similar to that in the supine position in our clinical practice, although the set-up time might be longer for patients with higher BMI. As robust results of daily image-guided set-up are not yet available, this study provides the first evidence of inter-fraction and intra-fraction set-up errors and margins for RSD breast radiotherapy.

## Conclusions

This study determined the inter-fraction set-up error and intra-fraction motion in RSD breast radiotherapy with free breathing, supporting the use of CBCT as an effective modality to evaluate and improve the accuracy of RSD breast radiotherapy. Inter-fraction set-up errors did not increase as a function of time during the course of radiotherapy. The magnitude of inter-fraction set-up error was correlated with high body weight and BMI. Our results suggest that CBCT might be beneficial to these patients for the detection of substantial set-up error, which could not have been detected if these patients were aligned by using skin marks alone. In addition, patients could maintain the RSD position with minimal baseline drift during treatment. The anisotropic PTV margins of 6.5, 10.2, and 8.9 mm in the RL, AP, and SI directions might be used for RSD breast radiotherapy without CBCT; margins of 4.7, 6.3, and 5.4 mm in the RL, AP, and SI directions might be used for those treated with rigorous daily CBCT scans.

## Abbreviations

ANOVA: Analysis of variance
AP: Anterior–posterior
BMI: Body mass index
CBCT: Cone-beam computed tomography
CTV: Clinical target volume
DIBH: Deep inspiration breath-hold
LAD: Left anterior descending coronary artery
PTV: Planning target volume
RL: Right–left
RSD: Reverse semi-decubitus
SD: Standard deviation
SI: Superior–inferior
